# Supplementary material for: Prediction of the spread of African swine fever through pig and carcass movements in Thailand using a network analysis and diffusion model
Source: PeerJ. 2023 May 9;11:e15359. doi: 10.7717/peerj.15359 (PMC10178211; doi:10.7717/peerj.15359)
Supplement: Supplemental Information 1 [file peerj-11-15359-s001.pptx]

## Slide 1
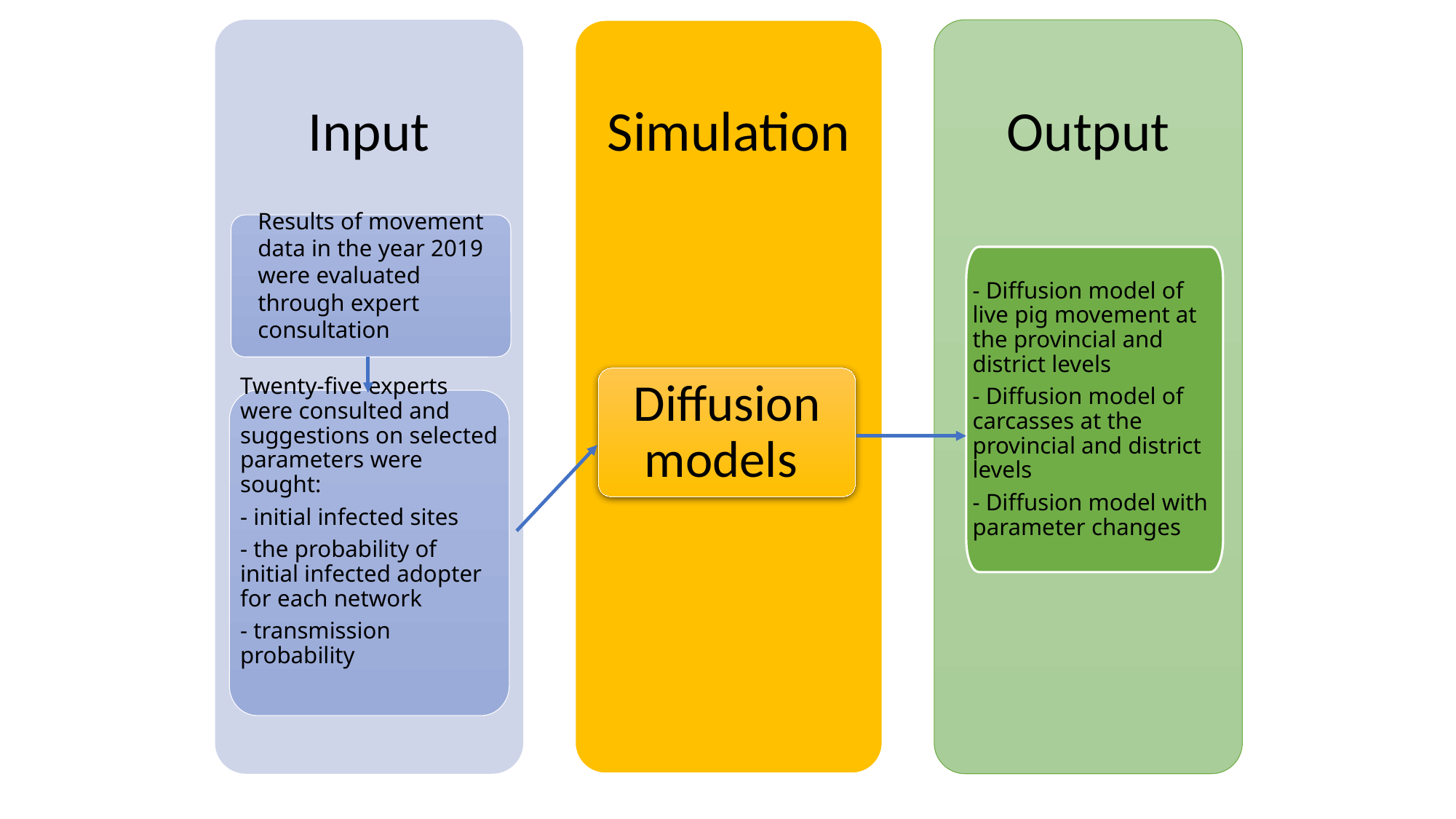

Input
Simulation
Output
- Diffusion model of live pig movement at the provincial and district levels
- Diffusion model of carcasses at the provincial and district levels
- Diffusion model with parameter changes
Diffusion models
Twenty-five experts were consulted and suggestions on selected parameters were sought:
- initial infected sites
- the probability of initial infected adopter for each network
- transmission probability
Results of movement data in the year 2019 were evaluated through expert consultation
