# Supplementary material for: Prediction of the spread of African swine fever through pig and carcass movements in Thailand using a network analysis and diffusion model
Source: PeerJ. 2023 May 9;11:e15359. doi: 10.7717/peerj.15359 (PMC10178211; doi:10.7717/peerj.15359)
Supplement: Supplemental Information 5 [file peerj-11-15359-s005.docx]

**Questionnaire for African Swine Fever (ASF) in Thailand**

**(Please note that original version is in Thai)**

**List of questions**

1. Please provide your e-mail address.

…………………………………………………………………………………………………………………………..

2. Please specify the job that you are engaged in.

…………………………………………………………………………………………………………………………..

3. Please specify the number of years that you have worked with pigs.

…………………………………………………………………………………………………………………………..

4. Please specify the province where you currently work. If there are multiple provinces, specify the province where you are most engaged.

…………………………………………………………………………………………………………………………..

5. Does the province where you work have wild boar?

…………………………………………………………………………………………………………………………..

6. Is there a risk of ASF in the province you work?

…………………………………………………………………………………………………………………………..

7.. Which pig farm is at the highest risk for ASF?

…………………………………………………………………………………………………………………………..

8. Has your province ever been suspected of ASF?

…………………………………………………………………………………………………………………………..

9. Do you have any illegal movements of carcasses and/or live pigs in your province?

…………………………………………………………………………………………………………………………..

10. Do you think that there is an outbreak of ASF in Thailand? If yes, what are the risks?

…………………………………………………………………………………………………………………………..

11. Which detection methods can provide early signs of an outbreak? Why?

…………………………………………………………………………………………………………………………..

12. What are the effective measures for preventing ASF?

…………………………………………………………………………………………………………………………..

13. If an ASF outbreak occurs in Thailand, which actions must be taken immediately?

…………………………………………………………………………………………………………………………..

14. Do you think the biosecurity measures of commercial pig farms are sufficient to prevent ASF?

…………………………………………………………………………………………………………………………..

15. Are there places associated with pig husbandry that may cause ASF?

…………………………………………………………………………………………………………………………..

16. Have you seen a disease carrier such as a tick in the Ornithodoros genus, in your province? (illustrated below)

…………………………………………………………………………………………………………………………..

17. Have you seen the introduction of infected animals or those suspected of being infected with ASF, in your province?

…………………………………………………………………………………………………….…………………….

18. Have you seen the introduction of infected/contaminated/suspected products associated with ASF in your province?

…………………………………………………………………………………………………………………………..

19. Do you think the experience of the pig farmer is sufficient to prevent ASF?

…………………………………………………………………………………………………………………………..

20. Have you seen pig traders in your province who are at risk of spreading the disease?

…………………………………………………………………………………………………………………………..

21. Have you seen a live pig market in your province?

…………………………………………………………………………………………………………………………..

22. Have you seen a slaughterhouse in your province?

…………………………………………………………………………………………………………………………..

23. Do you agree to having all your responses published anonymously?

…………………………………………………………………………………………………………………………..

**************
