# Supplementary material for: Prediction of the spread of African swine fever through pig and carcass movements in Thailand using a network analysis and diffusion model
Source: PeerJ. 2023 May 9;11:e15359. doi: 10.7717/peerj.15359 (PMC10178211; doi:10.7717/peerj.15359)
Supplement: Supplemental Information 6 [file peerj-11-15359-s006.pdf]

คำถามสำหรับโรค African Swine Fever

รายการคำถาม

1. ชื่อผู้ใช้ (e-mail)

....

2. โปรดระบุบทบาทของท่าน

....

3. ประสบการณ์ในการทำงานเกี่ยวกับสุกร (โปรดระบุตัวเลขเป็นจำนวนปี)

....

4. จังหวัดที่ท่านทำงานเกี่ยวกับสุกรในปัจจุบัน หากมีหลายจังหวัดโปรดระบุจังหวัดที่ท่านเกี่ยวข้องมากที่สุด

....

5. จังหวัดที่ท่านอยู่มีหมูป่าหรือไม่

....

6. จังหวัดที่ท่านอยู่ มีความเสี่ยงต่อการเกิดโรคอหิวาต์แอฟริกาในสุกร เพียงใด

....

7. ฟาร์มสุกรแบบใดที่มีความเสี่ยงต่อการเกิดโรคอหิวาต์แอฟริกาในสุกร

....

8. ปัจจุบัน ท่านคิดว่าในประเทศไทยมีการระบาดของโรคอหิวาต์แอฟริกาในสุกรหรือไม่ หากมีคาดว่าเป็นพื้นที่จังหวัดใด และปัจจัยเสี่ยงคืออะไร

....

9. ในจังหวัดท่านมีการลักลอบขนหรือเคลื่อนย้ายสัตว์หรือไม่ (รวมซากและผลิตภัณฑ์ด้วย)

....

10. จังหวัดของท่านเคยสงสัยว่ามีการเกิดโรคอหิวาต์แอฟริกาในสุกร หรือไม่

....

11. วิธีการตรวจวิธีใดเหมาะสมสำหรับการตรวจหาโรค

....

12. มาตรการที่มีประสิทธิภาพในการป้องกันการเกิดโรคอหิวาต์แอฟริกาในสุกร ในประเทศไทยคือ

....

13. หากเกิดการระบาดของโรคอหิวาต์แอฟริกาในสุกร มาตรการใดเป็นมาตรการที่ควรปฏิบัติ (อาจมีหลายมาตรการได้)

....

14. ท่านคิดว่ามาตรการความปลอดภัยทางชีวภาพของฟาร์มสุกรแบบธุรกิจ มีความเพียงพอต่อการป้องกันการเกิดโรคหรือไม่

....

15. ฟาร์มหรือสถานที่ที่เกี่ยวกับสุกรใด มีความเสี่ยงในการเกิดโรคมากที่สุด

....

16. ในจังหวัดของท่าน เคยพบพาหนะนำโรค เช่น เห็บในสกุล Ornithodoros หรือไม่ (ภาพประกอบด้านล่าง)

....

17. ในจังหวัดของท่าน เคยนำเข้าสัตว์ติดเชื้อหรือสงสัยว่าติดเชื้อโรคอหิวาต์แอฟริกาในสุกร หรือไม่

....

18. ในจังหวัดของท่าน เคยนำเข้าผลิตภัณฑ์ที่ปนเปื้อนเชื้อหรือสงสัยว่าติดเชื้อโรคอหิวาต์แอฟริกาในสุกร หรือไม่

....

19. ท่านคิดว่าประสบการณ์ของเจ้าของฟาร์มสุกรมีความสัมพันธ์กับการป้องกันโรคอหิวาต์แอฟริกาในสุกร หรือไม่

....

20. นจังหวัดท่านมีผู้รับซื้อและขายสุกร (trader) ที่มีความเสี่ยงต่อการแพร่โรคระบาดหรือไม่

....

21. ในจังหวัดท่านมีตลาดค้าสุกรมีชีวิตหรือไม่

....

22. ในจังหวัดท่านมีโรงฆ่าหรือไม่

....

23. ท่านเห็นด้วยที่จะให้มีการเผยแพร่ข้อมูลงานวิจัยในภาพรวม โดยการเผยแพร่นี้ต้องไม่สามารถตรวจสอบย้อยกลับ เพื่อระบุตัวตนของท่านได้

....

****
